# Chronic Pyruvate Supplementation Increases Exploratory Activity and Brain Energy Reserves in Young and Middle-Aged Mice

**DOI:** 10.3389/fnagi.2016.00041

**Published:** 2016-03-16

**Authors:** Hennariikka Koivisto, Henri Leinonen, Mari Puurula, Hani Sayed Hafez, Glenda Alquicer Barrera, Malin H. Stridh, Helle S. Waagepetersen, Mika Tiainen, Pasi Soininen, Yuri Zilberter, Heikki Tanila

**Affiliations:** ^1^A. I. Virtanen Institute, University of Eastern Finland, Kuopio, Finland; ^2^Biology Department, Faculty of Science, Suez University, Suez, Egypt; ^3^Institute of Physiology, Czech Academy of Sciences, v. v. i., Prague, Czech Republic; ^4^Faculty of Health and Medical Sciences, University of Copenhagen, Copenhagen, Denmark; ^5^NMR Metabolomics Laboratory, School of Pharmacy, University of Eastern Finland, Kuopio, Finland; ^6^Institut national de la santé et de la recherche médicale UMR_S 1106, Institut de Neurosciences des Systèmes, Aix-Marseille Université, Marseille, France

**Keywords:** Alzheimer’s disease, aging, memory, explorative activity, glycogen

## Abstract

Numerous studies have reported neuroprotective effects of pyruvate when given in systemic injections. Impaired glucose uptake and metabolism are found in Alzheimer’s disease (AD) and in AD mouse models. We tested whether dietary pyruvate supplementation is able to provide added energy supply to brain and thereby attenuate aging- or AD-related cognitive impairment. Mice received ~800 mg/kg/day Na-pyruvate in their chow for 2–6 months. In middle-aged wild-type mice and in 6.5-month-old APP/PS1 mice, pyruvate facilitated spatial learning and increased exploration of a novel odor. However, in passive avoidance task for fear memory, the treatment group was clearly impaired. Independent of age, long-term pyruvate increased explorative behavior, which likely explains the paradoxical impairment in passive avoidance. We also assessed pyruvate effects on body weight, muscle force, and endurance, and found no effects. Metabolic postmortem assays revealed increased energy compounds in nuclear magnetic resonance spectroscopy as well as increased brain glycogen storages in the pyruvate group. Pyruvate supplementation may counteract aging-related behavioral impairment, but its beneficial effect seems related to increased explorative activity rather than direct memory enhancement.

## Introduction

One characteristic metabolic change with aging is a blunted glucose response to stress, whether physical, emotional, or cognitive (Gold, 2005). This can be demonstrated as a mismatch between adrenaline (epinephrine) release and subsequent rise in blood glucose levels in aged rats compared to young ones (Gold, 2005). On the one hand, the inability to maintain adequate blood glucose levels upon challenge may lead to insufficient supply of glucose to the brain and cognitive impairment (McNay, 2005). On the other hand, acute glucose infusion has been shown to improve cognitive performance in both rats and humans, especially during a highly demanding (due to difficulty or duration) task (Gold, 2005). The aging-related susceptibility to episodes with insufficient brain glucose supply is further exacerbated by common age-associated conditions, such as treatment of type 2 diabetes with glucose lowering medications or Alzheimer’s disease (AD) associated with reduced levels of glucose transporter 1 and 3 (Liu et al., 2008).

The brain has also a buffer system to counteract sudden decrease in glucose supply. The most important mechanism is glycogen storage in astrocytes (Bélanger et al., 2011), although a recent study suggests that neurons are also capable of storing glycogen (Saez et al., 2014). Recent experimental evidence has established that glycogenolysis in astrocytes is instrumental for some key functions of astrocytes. First, despite efficient uptake of neurotransmitter glutamate by GLT-1 transporter into astrocytes, about 15% of glutamate under this shuttle is used for energy and thus needs to be replenished. This is largely done by pyruvate carboxylase leading to oxaloacetate with further conversion to α-ketoglurate, which then leaves the tricarboxylic acid cycle (TCA) for conversion to glutamate (Hertz et al., 2015). The process is largely abolished by inhibition of glycogenolysis (Hertz et al., 2015). Second, accumulating evidence indicates that the energy for Na^+^K^+^-ATPase-mediated K^+^ uptake derives from astrocytic glycogenolysis (Hertz et al., 2015). Through these two mechanisms, astrocytic glycogen stores may play a pivotal role in controlling excitability of neighboring neurons. This propensity may explain the findings that inhibition of glycogenolysis impairs memory consolidation in neonatal chicks (Gibbs et al., 2007) and rats (Suzuki et al., 2011). Notably, astrocytic glycogen stores are highly dynamic and susceptible to depletion upon physiological challenge. For instance, one-night sleep deprivation in rats leads to ~40% depletion of brain glycogen levels, which recover after 15 h of sleep (Kong et al., 2002). Thus, poor night sleep, a common nuisance at old age and especially AD, may lead to depleted astrocytic glycogen stores and thereby further impair memory.

Neurons are potentially capable of utilizing interstitial lactate as an alternate energy substrate to glucose (Schurr et al., 1988; Amaral, 2013; Ivanov et al., 2014), since even at rest conditions, the interstitial fluid in neocortex and hippocampus contains about 2–3 mM of lactate (Zilberter et al., 2010). However, recent direct *in vivo* measurements in freely moving rats revealed that glucose is preferentially utilized by neurons (Lundgaard et al., 2015), suggesting that lactate may be consumed under conditions of extreme energy demands (Dienel, 2012). Whereas pyruvate is the end product of glycolysis and thus a direct energy substrate for mitochondria, lactate needs to be first converted to pyruvate. The latter reaction depends on the availability of cytoplasmic NAD^+^, which may be compromised under metabolic crisis (Zilberter et al., 2015). Pyruvate, and to a lesser extent lactate, incubation prior to glucose deprivation sustained synaptic and metabolic function in hippocampal slices in one study (Shetty et al., 2012). Moreover, pyruvate exposure led to the enhancement of glycogen stores with time, compared to glucose alone. Radiolabeled pyruvate given as a bolus injection could pass the blood–brain barrier (BBB) similarly to glucose and was detected in a large amount in the brain 5 min after the injection (Miller and Oldendorf, 1986; Gonzalez et al., 2005).

Multiple neuroprotective effects of pyruvate after systemic administration have been reported in animal models in the cases of brain injury (Fukushima et al., 2009), ischemia (Kim et al., 2005; Yi et al., 2007), glutamate neurotoxicity (Miao et al., 2011), hemorrhagic shock (Mongan et al., 2003; Su et al., 2013), hydrogen peroxide-induced cell death (Nakamichi et al., 2005), oxygen-glucose deprivation (Ryou et al., 2013), cognitive impairment due to hypoglycemia (Suh et al., 2005), ethanol-induced neurodegeneration (Ullah et al., 2013), and zinc-induced cortical neuronal death (Sheline et al., 2000). In addition, a recent study reported improved spatial memory in the Morris swim task in both 6- and 12-month-old 3xTg AD model mice after repeated i.p. pyruvate injections over months (Isopi et al., 2015).

We have recently reported that 5-week dietary supplementation with pyruvate + β-hydroxybutyrate in the chow reversed impaired tolerance of APPswe/PS1dE9 mice to hypoglycemia, normalized their reduced brain glycogen stores, and attenuated their enhanced neuronal excitability both *in vitro* and *in vivo* (Zilberter et al., 2013). Our subsequent pilot studies showed that a similar effect can be obtained by pyruvate supplementation alone. These findings raised the question whether oral pyruvate supplementation could also provide a means to counteract aging and AD-related memory impairment as alluded to by the study in 3xTg mice (Isopi et al., 2015). However, the previous study used only one behavioral assay (the Morris swim task) and only reported the number of platform crossings and latency in the probe trial without commenting eventual pyruvate effects on non-cognitive factors, such as swimming speed that may affect learning. Since the existing literature on pyruvate mainly focuses on its effects on muscle force and energy metabolism, we wanted to study not only cognitive effects of pyruvate but also its impact on non-cognitive factors, such as spontaneous activity, muscle force, endurance, and anxiety. To this end, we ran three separate experiments with a large behavioral test battery and assessed the direct effects of pyruvate substitution on the indices of brain energy metabolism. The first experiment was run in middle-aged wild-type mice and the second one in adult APPswe/PS1dE9 (APP/PS1) mice that both show only modest memory impairment compared to young adult mice (Minkeviciene et al., 2008). The third experiment addressing alternative non-cognitive mechanisms of pyruvate action was done in adult wild-type mice owing to the limited availability of older wild-type or APP/PS1 mice and to clarify the observed consistency of pyruvate effects independent of age.

## Materials and Methods

### Animals

*Experiment 1* comprised 36 male C57Bl/6J mice (breeding at Laboratory Animal Center, University of Eastern Finland, Kuopio, Finland) that started the dietary intervention at 6 months, behavioral testing at 12 months of age, and were euthanized when reaching 13 months. *Experiment 2* comprised 25 male APPswe/PS1dE9 transgenic mice (Jankowsky et al., 2004) that started the dietary intervention at 4.5 months, behavioral testing at 6.5 months of age, and were euthanized at the age of 7 months. *Experiment 3* comprised 20 male C57Bl/6J mice that started the dietary intervention at 3 months, behavioral testing at 5 months of age, and were euthanized at 6.5 months of age. In addition, another group of 19 male C57Bl/6J mice were tested with acute pyruvate vs. saline injection at the age of 5 months. The mice came from a local breeding colony at University of Eastern Finland that was based on breeder mice from Johns Hopkins University (Baltimore, MD, USA). The line was originally generated as C3H × C57Bl/6J hybrid but had been back-crossed to C57Bl/6J strain for 18 generations.

The animals were group-housed until the behavioral tests in controlled environment (temperature 22 ± 1°C, light 0700–1900 hours, and humidity 50–60%), and food and water were freely available. All behavioral tests were conducted during the light phase. The experiments were conducted according to the Council of Europe (Directive 86/609) and Finnish guidelines and approved by the Animal Experiment Board of Finland.

### Treatment

#### Chronic Pyruvate Administration

The test group (PYR) received experimental chow supplemented with 0.6% (w) of Na-pyruvate (Safe Diets, Augy, France). The control group (STD) received the same basic rodent chow (A04, Safe Diets). With the average food intake of 4 g, this corresponds to 800 mg of pyruvate/day, which is at the upper range of effective pyruvate doses in earlier *in vivo* studies (Suh et al., 2005; Fukushima et al., 2009; Isopi et al., 2015).

#### Acute Pyruvate Administration

The mice received Na-pyruvate (Sigma, St. Louis, MO, USA) 500 mg/kg i.p. or the same molar concentration of NaCl (260 mg/kg i.p.). This single dose affords neuroprotection against cortical concussion injury and increases brain glucose and pyruvate levels as measured by *in vivo* microdialysis (Fukushima et al., 2009). All cage labels about the treatment groups were coded so that the researchers running behavioral tests or assays on postmortem samples were blinded as to the treatment.

### Behavioral Testing

#### Morris Swim Task (Water Maze)

Spatial learning and memory was tested with Morris swim navigation task. The apparatus consisted of a white plastic pool with diameter of 120 cm and of transparent platform (10 cm × 10 cm) submerged 1.0 cm below the water surface. Water temperature was kept at 20 ± 5°C throughout the testing, and 8–10 min recovery period in a warmed cage was allowed between all trials. Before actual navigation task, the mice were pretrained (2 days) to find and climb onto the submerged platform, aided by a guiding alley (1 m × 14 cm × 25 cm) with opaque walls preventing any spatial clues to be seen. In the learning phase (days 1–4), five 60-s trials per day were conducted with the hidden platform. The platform location was kept constant and the starting position varied between four constant locations at the pool rim, with all mice starting from the same position and nose pointing toward the wall in any single trial. If a mouse failed to find the escape platform within 60 s, it was placed on the platform for 10 s by the experimenter. Also, when mice found the platform independently, they were allowed to stay on the platform for 10 s. On day 5, the last trial (fifth) was run without the platform to test the search bias that was followed for 60 s. The experimenter marked the start of each trial using a remote controller and the trial ended automatically when a mouse landed on the platform. Swim paths and other parameters were tracked and recorded by video tracking system EthoVision XT 7 (Noldus, Wageningen, Netherlands).

Wall-swimming tendency (thigmotaxis) was assessed by calculating the time the mouse spent in the outer zone within 10 cm of the wall. The search bias during the probe trial was measured by calculating the time the mice spent in the vicinity of the former platform position. We defined this as a target area, centered on the platform, with a diameter of 30 cm. This target area comprised 6.25% of the total surface, which means that a mouse swimming randomly in the pool would be expected to spend 3.75 s in the target area during the 60-s probe trial.

#### Odor Recognition Task

The task is based on individual specific odors of mice. Before the test, each mouse to be tested got two small (diameter 20 mm) odorless balls made of birch wood (Step Systems Oy, Lahti, Finland) on the cage bedding. Similarly, a specifically assigned odor donor mouse got several of these balls. These were left overnight to get impregnated with the mouse odor, and in the case of the test mice to let them get adopted to the presence of the balls in their home cage. The wooden balls were removed in the morning. After 2–4 h, the mouse was presented with two balls, one impregnated with its own odor and the other one from the cage of the donor mouse. The test was replicated the next day with one ball of the mouse’s own odor and a second ball impregnated with the odor of another unfamiliar mouse. We measured the total time sniffing (the nose pointing to the ball at a distance <2 cm) during a 120-s test session. Only if the total sniffing time exceeded 10 s, we also counted the odor preference as the percent of total time that the mouse was sniffing the ball with unfamiliar odor.

#### Passive Avoidance

The test was conducted in a shuttle box (*L* 44 cm × *W* 17 cm × *H* 25 cm; Med Associates Inc., St. Albans, VT, USA) that was divided by a partition into two halves. One half was open and well-lit, while the other half was closed by black plastic walls and cover plate. Both compartments had a similar grid floor. The partition had an arch-shaped 6 cm wide opening that could be closed by a slide door. On day 1, the mouse got first 5 min to freely explore both compartments. Then, the mouse was placed in the open compartment and the slide door was closed for 30 s. Once the door was opened, the experimenter took the time for the mouse to enter the dark compartment, closed the opening, and delivered two mild foot shocks (0.30 mA, 2 s, interval 2 s). After the foot shock, the mouse was immediately returned to its home cage. On day 2, the time to enter the dark compartment was measured with a stop watch until a cutoff time of 180 s. No shocks were delivered on day 2.

#### Spontaneous Explorative Activity

Spontaneous explorative activity was assessed by using an automated activity monitor (TruScan, Coulbourn Instruments, Whitehall, PA, USA) based on infrared photo beam detection. The system consisted of an observation cage with white plastic walls (26 cm × 26 cm × 39 cm) and two frames of photo detectors enabling separate monitoring of horizontal (XY-move time) and vertical activity (rearing). The test cage was cleaned with 70% ethanol before each mouse to avoid odor traces. The following parameters were measured during a 10-min session: ambulatory distance (gross horizontal locomotion) and rearing time. The recording was repeated 48 h later to assess habituation to the environment.

#### Marble Burying Test

To assess neophobia and general housekeeping activity, the home cage floor was filled with double amount of clean bedding material and nine glass marbles (diameter 1 cm) were inserted in a 3 × 3 array onto the bedding. Visible marbles were then counted after 24 h.

#### Elevated Plus Maze

The maze, as the name implies, had a shape like a plus sign and consisted of a square platform (5 cm × 5 cm) and four arms (30 cm × 5 cm, two open and two closed), all made of black plastic but covered with white plastic to help video monitoring. The maze platform was 50 cm above the floor in a dimly lit room. Each mouse was placed on the center platform facing an open arm and allowed to explore the maze for 5 min. A video camera hanging from the ceiling recorded the session, which was analyzed offline with EthoVision XT 7 software. The time spent in the open and closed arms, the total distance, and speed in 30 s bins were calculated as well as the percentage of total time spent on the open arms to index anxiety. Only mice making more than five moves between arms during the test were included in the statistical analysis for % time on the open arms.

#### Open Field

The test was conducted in the dry pool (diameter 120 cm) used for Morris swim task. The mouse was placed in the arena center and its movement was recorded for 10 min. Using EthoVision XT 7 software, we calculated the total distance traversed, speed in 30-s bins, and the % time spend in the arena center (diameter 40 cm).

#### Pain Threshold

The test was conducted 2 weeks after the passive avoidance test in the same apparatus. The mouse was first given 3 min to fully familiarize with the environment and calm down. Then, electric shocks were delivered every 30 s with gradually increasing intensity (0.05, 0.06, 0.08, 0.10, 0.12, 0.15, 0.2, and 0.3 mA) until the mouse reacted by jumping. Before that, the current intensity eliciting a startle response was also recorded.

#### Grip Force Test

The grip force was measure using a simple spring scale (Kouluelektroniikka Oy, Rauma, Finland) with a metal grid for the mouse to grip with both front paws. The scale was positioned at a 45° angle at the edge of a table. The mouse was gently pulled by the tail until it could no longer withhold the grip. The test was repeated five times every 30 s and the best reading was recorded.

#### Treadmill Test

The apparatus was a treadmill designed for mice (LE8710R, Bioseb, France) with adjustable speed and inclination. The mouse was motivated to keep running by a gentle electric shock (0.15 mA during training and 0.2 mA during testing) at the proximal end of the belt. The mice were familiarized with the procedure in two 10-min sessions during 2 days. On the first day, the belt was rolling at a fixed speed of 15 cm/s; on the second day, the speed was increased gradually from 15 to 25 cm/s. On the test day, the speed of the belt was increased from 15 cm/s with a step of 5 cm/s every minute until the speed of 35 cm/s was reached. When the mouse took five electric shocks with 1-s intervals or made only minimum spurts to avoid the shocks, it was judged to be exhausted and the test was terminated.

### Metabolic Assays

#### Histochemical Assay – Periodic Acid Schiff Staining

In Experiment 1, after behavioral testing mice were deeply anesthetized with pentobarbiturate-chloral hydrate cocktail (60 mg/kg i.p. each) and perfused transcardially with 50-ml ice-cold saline (10 ml/min) for 5 min. The brain was removed and split sagittally. The left hemisphere was immersion fixed in 4% paraformaldehyde for 4 h, transferred to a 30% sucrose solution overnight, and stored in a cryoprotectant in −20°C for later histology. The hemisphere was cut on a freezing slide microtome into 20-μm coronal sections. Periodic acid schiff (PAS) staining was done on two adjacent sections at the level of mid-hippocampus (A −2.7 from bregma) in eight STD mice and eight PYR mice. First, the sections were hydrated to water and after that oxidized in 0.5% periodic acid solution for 5 min. After rinsing in distilled water, the sections were soaked in Schiff reagent for 1 min. To get right dark pink color, sections were washed in lukewarm water for 5 min. Dehydration was done with rising alcohol series and coverslipping with DePeX mounting medium. Sections were photographed using the Zeiss Imager M2 microscope (Zeiss, Oberkochen, Germany) and the attached AxioCam ERc 5s camera with a 2.5× objective. Images were transformed to grayscale in Photoshop CS6 software (Adobe Systems Inc., San Jose, CA, USA), which was also used for image analysis. The mean optic densities values (0 for black to 255 for white) were determined from four regions of interest: DG molecular layer, CA1 all layers, white matter (alveus + corpus callosum), and visual cortex.

#### Enzymatic Analysis of Glycogen Content

At the end of Experiment 3, five young adult mice on STD and five on PYR diet were euthanized with a microwave radiator (5 W, 0.85 s, Muromachi Kikai Co., Ltd., Tokyo, Japan) and the brains were removed, the cortex dissected out, and stored at –70°C. One STD mouse had to be discarded because of failed procedure. The quantitative amounts of glycogen in the tissue were assessed in a coupled enzymatic assay by measuring the production of NADPH in the conversion of glucose-6-phosphate to 6-phosphogluconolactone as described by Brown et al. (2003). To prepare the samples for the assay, the tissues were homogenized by sonication in ice-cold 70% ethanol. The samples were centrifuged at 20,000 × *g* for 10 min and the supernatant was discarded. The remaining pellet was washed twice with ethanol and once with water and resuspended in an acetate buffer (pH 5). Aliquots of the homogenized pellets were adjusted to pH 2 by the addition of HCl. Amyloglucosidase dissolved in an acetate buffer (pH 5) was added to the acidified homogenates before incubation at 37°C and 50 rpm for 1 h in an alkaline solution (37.1 mM Tris base, 0.007% sodium azide, and 13.8 mM HCl) to ensure pH 7–8, i.e., the pH optimum for hexokinase and glucose-6-phosphate dehydrogenase. The reaction was initiated by the addition of hexokinase and glucose-6-phosphate dehydrogenase to final concentrations of 1.52 and 0.54 U/ml, respectively. The fluorescence was measured before addition of the enzymes and following 40 min of incubation at room temperature, using 360 and 415 nm as excitation and emission wavelengths, respectively. Glucose in the concentration range of 20–200 μM was used as a standard, and the amount of glycogen in the samples is expressed as nanomoles of glucose per milligram of protein. The protein amounts in the samples were determined using the BCA method employing bovine serum albumin as standard.

#### ^1^H NMR Analysis

After behavioral tests, mice of Experiment 2 (APP/PS1 mice at 7 months of age) were euthanized with cervical dislocation, the brain was rapidly removed, frontal 1/3 was cut off and snap frozen in liquid nitrogen, and stored at –70°C. We chose a frontal cut instead of hippocampal preparation to keep the postmortem delay to the minimum (now 80–100 s). Weighed tissue samples were subjected to perchloric acid (PCA) extraction. The frozen tissue samples were transferred into a precooled mortar and grounded by a pestle with liquid nitrogen to fine powder. The ground tissue powder was transferred into a cold 2-ml Eppendorf tube, and 1.0 ml of cold 0.9M PCA was added. The obtained suspension was homogenized by vortex mixing and sonication (15 min indirect sonication) in a water/ice bath. The supernatant was separated after centrifugation (10 min at 16,000 × *g* + 4°C) and neutralized using 2M potassium hydroxide. Precipitation (potassium perchlorate) was removed by centrifugation (15 min at 16,000 × *g* + 4°C) and the obtained supernatant was freeze-dried. Prior to the NMR measurements, neutralized, freeze-dried PCA extracts were dissolved to deuterium oxide containing 200 μM sodium 3-(trimethylsilyl)propionate-2,2,3,3-d4 (TSP) as a reference substance.

^1^H NMR spectra of the extracted samples were recorded on a Bruker Avance III HD 600 NMR spectrometer operating at 600.28 MHz and equipped with a Prodigy TCI 5 mm cryogenically cooled probe head. Standard 1D ^1^H NMR spectra were recorded with 64k data points using 32 transients and applying a standard Bruker zg pulse sequence. The acquisition time was 4.3 s and the relaxation delay 5.7 s. The spectra were measured at 295 K. The free induction decays (FIDs) were multiplied by an exponential window function with a 0.3-Hz line broadening. Identification of the metabolite signals was based on the data in the literature (Govindaraju et al., 2000; de Graaf et al., 2011) or in-house databases and spiking experiments. The quantification of the identified metabolites was done using quantitative quantum mechanical spectral analysis (qQMSA) approach with the program qQMTLS (Tiainen et al., 2014). The concentrations are reported as nanomole per milligram (wet tissue weight).

### Statistics

All statistics were calculated using IBM SPSS Statistics 19 software (IBM Corporation, NY, USA). Behavioral tests that were repeated on two or more days were analyzed by ANOVA for repeated measures using the diet as between-subject factor. Otherwise, the comparison of the diet groups was done with Student’s *t*-test, with the exception of passive avoidance for young animals where more than half of the animals reached the cutoff time. Here, non-parametric Mann–Whitney test was applied. Data are expressed as group mean ± SEM. Threshold for statistical significance was set at *p* < 0.05.

## Results

### Experiment 1: Chronic Pyruvate Supplementation in Middle-Aged Wild-Type Mice

#### Improved Spatial Learning after Chronic Pyruvate

**Figure 1:**
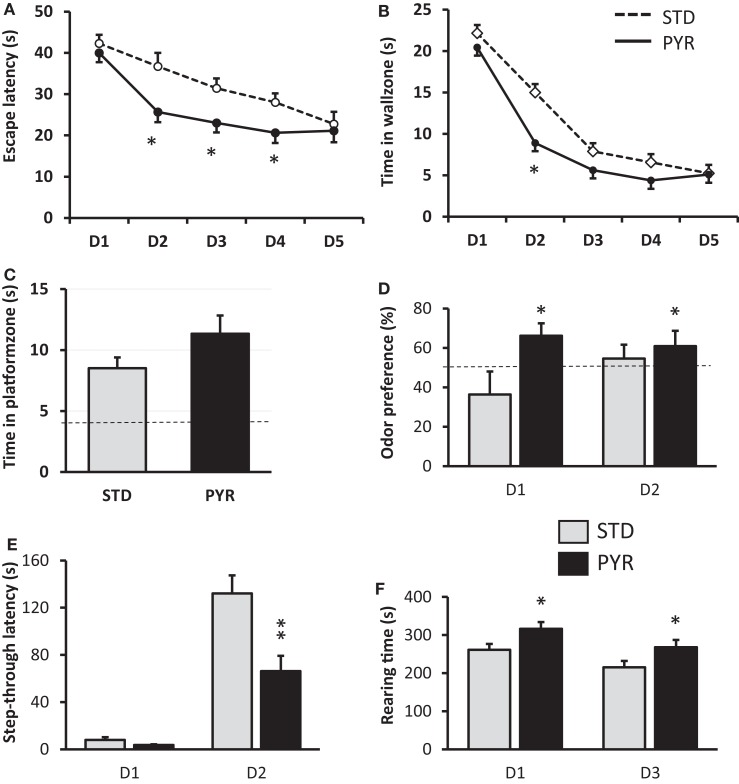
**Behavioral studies in 12-month-old wild-type mice treated with standard chow (STD) or pyruvate-supplemented chow (PYR) for 6 months**. **(A)** Escape latency during 5 days of Morris swim task acquisition. PYR group differs from STD group on this day (*p < 0.05, *t*-test). **(B)** Time in the wall zone (10 cm wide). PYR group differs from STD group on this day (*p < 0.05, *t*-test). **(C)** Time in the platform zone (15 cm radius from the former platform center) during the probe test on day 5. **(D)** Time preference to explore the odor of an unknown mouse over one’s own odor on two test days. The dashed line indicates chance level (50%). PYR group differs from STD group across days (*p < 0.05, ANOVA-RM). **(E)** Step-through latency in the passive avoidance task, cutoff 180 s. Day 1 is the training day and day 2 the test day after fear conditioning. PYR group differs from STD group on day 2 (**p < 0.01, *t*-test). **(F)** Rearing time in the transparent novel test cage on days 1 and 3, test duration 10 min of each day. PYR group differs from STD group across days (*p < 0.05, ANOVA-RM). Group Means ± SEMs are shown.

At 12 months of age (and after 6 months on test diet), spatial learning was tested in the Morris swim task. Mice on pyruvate supplementation (PYR) acquired the task faster than mice on standard chow (STD) as evidenced by shorter escape latency across five test days (*F*_1,34_ = 7.3, *p* = 0.01; Figure 1A). PYR mice were also faster to quit the inefficient strategy to search for an escape through the pool wall (thigmotaxis) than STD mice (*F*_1,34_ = 4.6, *p* = 0.04; Figure 1B). Neither measure showed a diet × day interaction (escape latency, *p* = 0.15; thigmotaxis, *p* = 0.36). The last swim on day 5 was a probe trial without the platform to assess the spatial search bias. PYR mice tended to spend more time in the vicinity of the former platform position that STD mice, but the difference did not reach statistical significance (*t*_34_ = 1.6, *p* = 0.11; Figure 1C).

Improved task acquisition, but only a modest augmentation, of memory retention can imply faster learning but also some non-cognitive effect of pyruvate supplementation, such as enhanced endurance in the swim task that is physically strenuous. To control this possibility, we compared swimming speeds between the groups. There was no overall difference in swimming speed across days (*F*_1,34_ = 0.5, *p* = 0.48). We also looked for the possibility of less fatigue on repeated trials in the PYR group, but a separate analysis of swimming speeds on the last trial of the four task acquisition days did not differ between the groups (*p* = 0.61).

#### Enhanced Odor Recognition but Impaired Fear Conditioning after Chronic Pyruvate

To assess the generalization of augmented learning effect of chronic pyruvate, the same 12-month-old mice were subjected to odor recognition and passive avoidance task. In the odor recognition task, the total sniffing time on day 1 was 45.8 ± 6.1 s (mean ± SEM) for STD mice and 57.1 ± 6.2 s for PYR mice, and on day 2, 42.3 ± 6.1 s for STD mice and 49.1 ± 6.0 s for PYR mice. There was no diet main effect on total sniffing time (*F*_1,34_ = 1.4, *p* = 0.24). However, the odor preference was significantly stronger across the two test sessions in PYR mice compared to STD mice (*F*_1,30_ = 4.7, *p* = 0.04; Figure 1D).

In the passive avoidance task, the groups did not differ in their latency to enter the dark compartment before conditioning (*t*_34_ = 1.8, *p* = 0.09), although mice in the PYR group tended to enter sooner than STD mice. However, after the fear conditioning, STD mice waited much longer than PYR mice before entering the dark compartment now associated with a foot shock (*t*_34_ = 3.3, *p* = 0.002; Figure 1E).

#### Increased Spatial Exploration but No Effect on Locomotion or Object Neophobia by Chronic Pyruvate

To better understand the ostensibly discrepant result of improved spatial learning and odor recognition but impaired retention of fear conditioning after chronic PYR administration, we ran a battery of control behavioral tasks to assess PYR effects on spontaneous exploration, locomotor activity, and object neophobia.

Although the ambulatory distance was slightly higher in the PYR group, the difference was not significant (*F*_1,34_ = 2.0, *p* = 0.17), and both groups showed robust reduction in the distance traversed during the second exposure to the novel test cage. In contrast, there was a significant difference in the rearing time between the groups (*F*_1,34_ = 6.4, *p* = 0.02), such that PYR mice spent more time rearing on both sessions (Figure 1F). Rearing is typically related to exploration of a new environment.

The marble burying test did not reveal significant differences in object neophobia between the diet groups. The STD mice left 5.0 ± 0.5 marbles out of nine visible and the PYR mice 4.8 ± 0.5 (*p* = 0.74).

### Experiment 2: Chronic Pyruvate Supplementation in 6.5-Month-Old APP/PS1 Mice

#### Modest Improvement in Spatial Learning after Chronic Pyruvate

**Figure 2:**
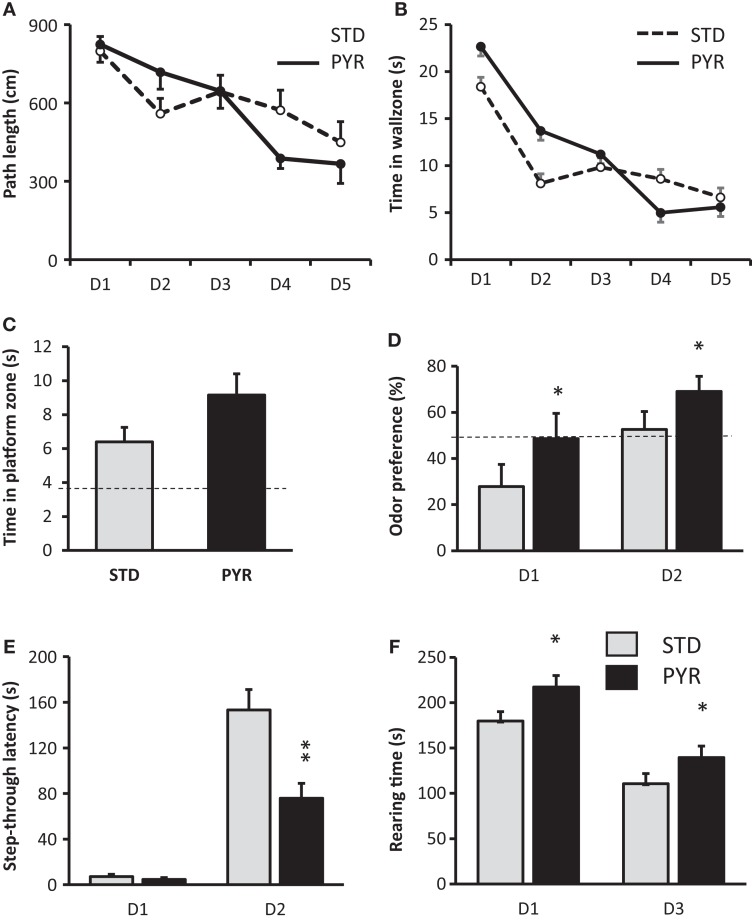
**Behavioral studies in 6.5-month-old APPswe/pS1dE9 mice treated with standard chow (STD) or pyruvate-supplemented chow (PYR) for 2 months**. **(A)** Escape latency during 5 days of Morris swim task acquisition. **(B)** Time in the wall zone (10 cm wide). **(C)** Time in the platform zone (15 cm radius from the former platform center) during the probe test on day 5. **(D)** Time preference to explore the odor of an unknown mouse over one’s own odor on two test days. The dashed line indicates chance level (50%). PYR group differs from STD group across days (*p < 0.05, ANOVA-RM). **(E)** Step-through latency in the passive avoidance task, cutoff 180 s. Day 1 is the training day and day 2 the test day after fear conditioning. PYR group differs from STD group on day 2 (**p < 0.01, *t*-test). **(F)** Rearing time in the transparent novel test cage on days 1 and 3, test duration 10 min of each day. PYR group differs from STD group across days (*p < 0.05, ANOVA-RM). Group Means ± SEMs are shown.

At 6.5 months of age (and 2 months on test diet), the diet groups differed significantly in their swimming speed across all test days (STD group faster; *F*_1,23_ = 7.1, *p* = 0.01; data not shown). Therefore, the task acquisition was assessed by the swim path length. The path length did not differ between the groups (*F*_1,23_ = 1.0, *p* = 0.76; Figure 2A). No overall difference was found in the time spent in the wall zone, either (*F*_1,23_ = 0.5, *p* = 0.48; Figure 2B). However, the diet × day interaction approached significance for the path length (*F*_4,92_ = 2.2, *p* = 0.08) and was significant for the time in wall zone (*F*_4,92_ = 2.6, *p* = 0.04; Figure 2B). In the probe test on day 5, PYR mice tended to swim more time in the vicinity of the former platform location (*t*_23_ = 1.8, *p* = 0.09; Figure 2C).

#### Enhanced Odor Recognition but Impaired Fear Conditioning after Chronic Pyruvate

In the odor recognition task, there was no diet main effect on total sniffing time (*F*_1,23_ = 0.1, *p* = 0.81). However, the odor preference was significantly stronger across the two test sessions in PYR mice compared to STD mice (*F*_1,18_ = 6.5, *p* = 0.02; Figure 2D). To be precise, on day 1, APP/PS1 mice on the STD diet actually showed aversion toward the odor of an unknown mouse, while showing no preference on day 2. In contrast, mice on PYR diet showed no preference on day 1 but a clear preference toward the novel odor on day 2 (Figure 2D).

In the passive avoidance task, the groups did not differ in their latency to enter the dark compartment before conditioning (*t*_23_ = 1.0, *p* = 0.35). However, after the fear conditioning, STD mice waited much longer than PYR mice before they entered the dark compartment now associated with a foot shock (*t*_23_ = 3.5, *p* = 0.002; Figure 2E).

#### Increased Spatial Exploration, No Effect on Locomotion but Decreased Object Neophobia by Chronic Pyruvate

The ambulatory distance did not differ between the diet groups (*F*_1,23_ = 0.3, *p* = 0.60), and both groups showed robust reduction in the distance traversed during the second exposure to the novel test cage. In contrast, there was a significant difference in the rearing time between the groups (*F*_1,23_ = 6.0, *p* = 0.02), such that PYR mice spent more time rearing on both sessions (Figure 2F). Rearing is typically related to the exploration of a new environment.

The pattern of behavioral effects of PYR was thus almost identical in 12-month-old wild-type mice and 6.5-month-old APP/PS1 mice. The only notable differences were the acquisition phase of Morris swim task, where 6.5-month-old mice on PYR did not show improvement, and marble burying test, in which APP/PS1 mice on PYR diet left more marbles visible than STD mice [STD: 2.8 ± 0.4 marbles; PYR: 4.6 ± 0.6 (*p* = 0.03)].

### Experiment 3: Pyruvate Supplementation in 5- to 6-Month-Old Wild-Type Mice

**Figure 3:**
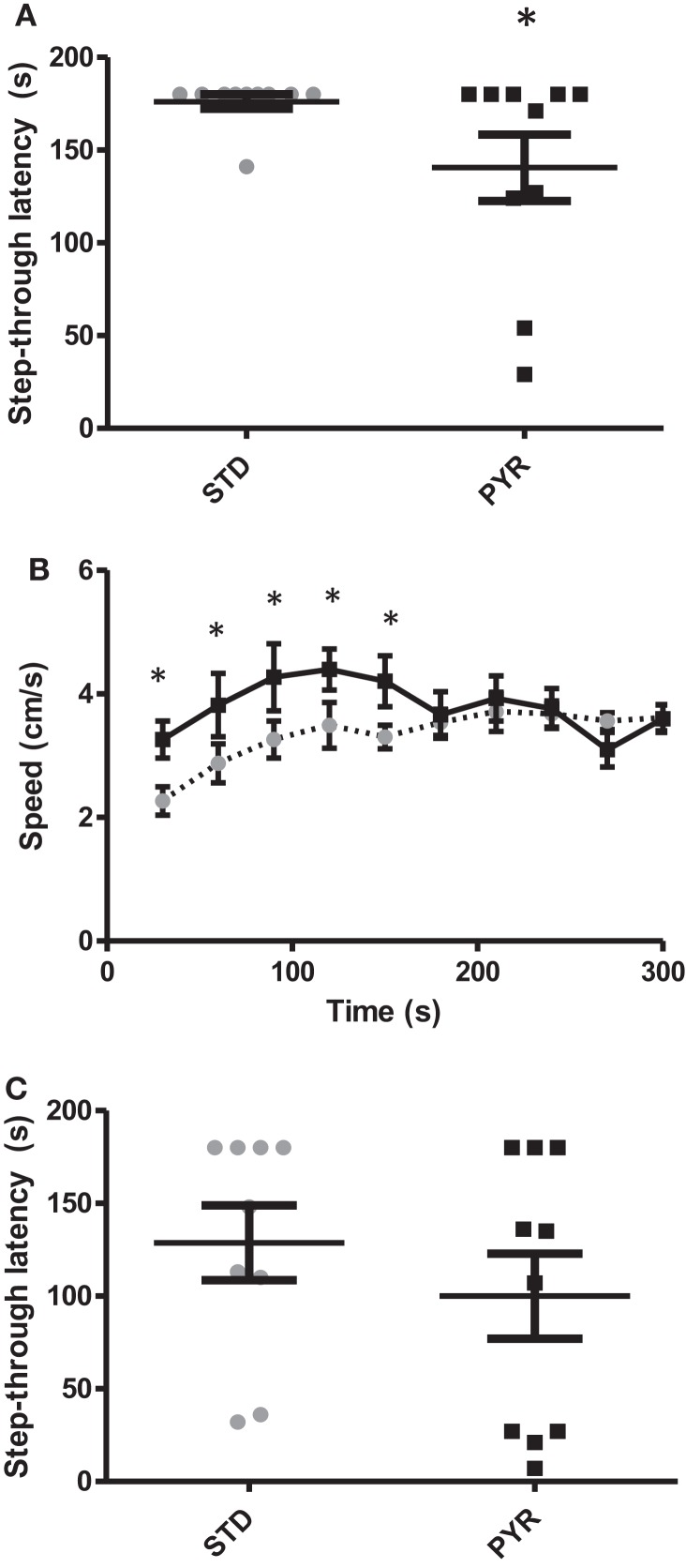
**Behavioral studies in young adult wild-type mice**. **(A)** Group means and individual values for step-through latency in the passive avoidance test run after chronic (2.5 months) dietary intervention. PYR group differs from STD group (*p < 0.05, Mann–Whitney *U*-test). **(B)** Running speed in the elevated plus maze plotted for each 30-s bin. During the first test half (2.5 min), mice on chronic PYR ran faster than STD mice (*p < 0.05, ANOVA-RM). **(C)** Group means and individual values for step-through latency in the passive avoidance test in the acute test groups. After the learning phase, half of the mice received Na-pyruvate at 500 mg/kg i.p., while other half received 260 mg/kg NaCl.

To shed light on the robust and reproducible finding that chronic PYR increased spatial learning and odor recognition but clearly impaired fear conditioning, we once again evaluated the possible non-cognitive effects of chronic PYR administration in young adult wild-type mice (the same C57Bl/6J background as in APP/PS1 mice). First, at the age of 5 months (and 2 months on test diet), we repeated the passive avoidance task. The diet groups did not differ in their latency to enter the dark compartment before conditioning (*t*_18_ = 0.3, *p* = 0.76). In contrast to middle-aged wild-type or adult APP/PS1 mice, all but one young adult wild-type mouse in the STD group waited until the cutoff time before entering the dark compartment. However, in the PYR group, half of the mice entered before the cutoff time (*p* < 0.05, Mann–Whitney *U*-test, Figure 3A). So, the chronic pyruvate effect was present also in young adult wild-type mice.

To rule out a pyruvate effect on nociception, we measured the minimum current for the foot shock to elicit startle and jump responses. There was no effect of the diet on either the startle response (STD: 0.086 ± 0.003 mA; PYR: 0.083 ± 0.005; *p* = 0.60) or the jump response (STD: 0.21 ± 0.02 mA; PYR: 0.24 ± 0.02; *p* = 0.32).

To assess the potential role of PYR on anxiety, we ran the standard elevated plus maze test. PYR mice moved a longer distance during the 5-min test than STD mice (STD: 9.9 ± 0.4 m; PYR: 11.9 ± 0.5 m; *t*_17_ = 3.3, *p* = 0.004). A closer analysis on the running speed revealed that PYR mice run at a higher velocity during the first 2.5 min (*F*_1,18_ = 6.0, *p* = 0.025), but then the running speed became even between the treatment groups (*p* = 0.97; Figure 3B). However, the % time spent on open arms, which is considered the strongest indicator of anxiety in this task, did not differ between the diet groups (STD: 21.6 ± 3.5%; PYR: 26.6 ± 4.5%; *t*_18_ = 0.9, *p* = 0.40). We also ran the open field test as an additional measure of anxiety. The relative time spend in the field center during 10 min did not differ between the treatment groups (STD: 4.7 ± 0.7%; PYR: 4.5 ± 0.8%; *t*_18_ = 0.2, *p* = 0.86). However, we could see the same pattern as in elevated plus maze: PYR mice moved faster than STD mice during the first 2.5 min (*F*_1,18_ = 4.3, *p* = 0.05), but then the difference in running speed disappeared (*p* = 0.27, 0.38, and 0.33 for the remaining quartiles).

To assess whether long-term pyruvate administration directly affects muscle function, we measured maximum grip force and endurance on a treadmill. There was no difference in the grip force (STD: 1103 ± 36 N; PYR: 1127 ± 41 N, *p* = 0.66) or the time until exhaustion on the treadmill (STD: 33.6 ± 6.7 min; PYR: 35.7 ± 6.1 min, *p* = 0.83).

Finally, we tested in another batch of young adult wild-type mice whether acute post-trial injection of Na-pyruvate (500 mg/kg i.p.) would also impair retention of fear conditioning in the passive avoidance task. There was no difference between the treatment groups either in the baseline latency (*t*_16_ = 0.5, *p* = 0.62) or latency 24 h after the fear conditioning (*t*_16_ = 1.0, *p* = 0.33; Figure 3C). This finding speaks against the possibility that PYR would have impaired memory consolidation.

#### Metabolic Effects of Chronic Pyruvate Supplementation

Among the middle-aged wild-type mice, the PYR group started with a lower body mass but gained more weight during the 7 months on special diet, resulting in a significant diet × age interaction (*F*_7,28_ = 5.0, *p* = 0.001). At the end of the study, the body weights for STD mice were 38.8 ± 1.0 g and for PYR mice 39.8 ± 0.9 g, *p* = 0.48. Similarly, among the young adult APP/PS1 mice, the PYR mice tended to gain more weight during 3 months on the special diet (*F*_2,22_ = 2.8, *p* = 0.08; end weight STD: 30.2 ± 0.6 g, PYR: 30.8 ± 0.7 g). On the other hand, no extra weight gain was observed in young adult wild-type mice on PYR diet (*F*_1,18_ = 0.1, *p* = 0.77).

**Figure 4:**
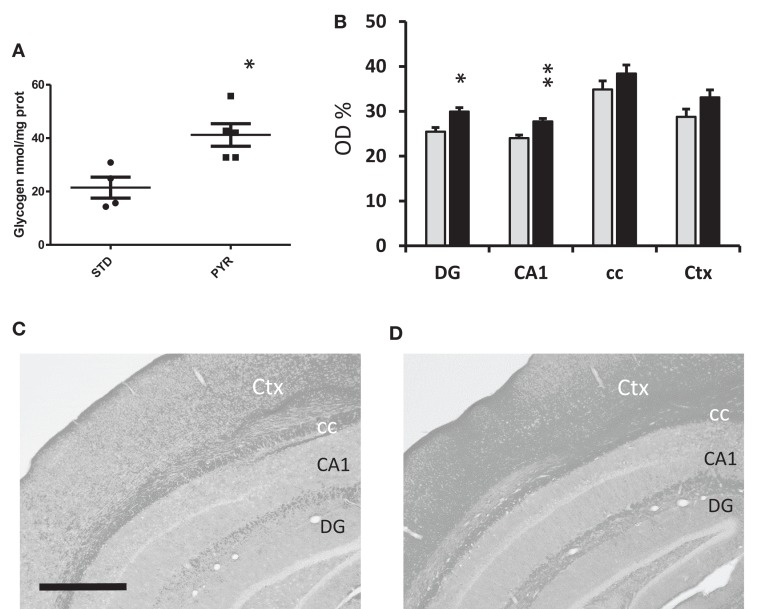
**Metabolic assessment of brain glycogen levels**. **(A)** Group means and individual values for cortical glycogen content in the enzymatic assay in young adult wild-type mice at 6 months of age after been on dietary intervention for 3 months by the time of euthanasia. PYR group has higher brain glycogen levels (*p < 0.05, *t*-test). **(B)** PAS staining for brain glycogen in 13-month-old wild-type mice been on the dietary intervention for 7 months. Data are given as relative optic density (OD%) with 0 corresponding to pure white and 100% to pure black. DG = dentate gyrus, molecular layer, CA1 = all layers of this subregion, cc = alveus + corpus callosum, and Ctx = all layers of visual cortex. PYR group has higher PAS staining intensity, *p < 0.05, **p < 0.01 (*t*-test). **(C,D)** Representative PAS-stained sections through the mid-hippocampus after conversion to grayscale. Scale bar = 500 μm. C = STD group and D = PYR group.

To further evaluate the impact of chronic PYR supplementation on brain energy reserves, we euthanized nine young adult wild-type mice of Experiment 3 with microwaves and measured the cortical glycogen content. It was higher in the PYR group than in the STD group (*t*_7_ = 3.3, *p* = 0.01; Figure 4A). We also stained brain sections from a subset of 16 middle-aged wild-type mice for PAS to visualize brain glycogen. The optic density for PAS was significantly higher in PYR mice than STD mice in the dentate gyrus (*p* = 0.01), CA1 (*p* = 0.005) but not in alveus/corpus callosum (*p* = 0.11) or overlying neocortex (*p* = 0.08) (Figures 4B–D).

**Figure 5:**
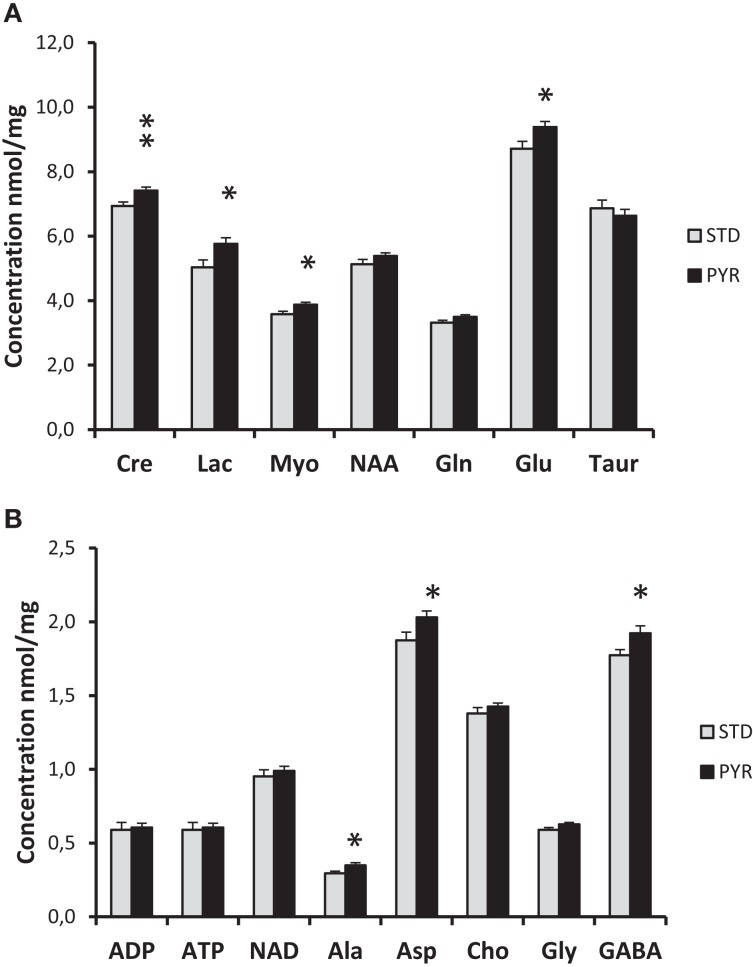
**^1^H-MRS analysis of snap frozen brains of 7-month-old APPswe/PS1dE9 mice been on the dietary intervention for 3 months by the time of euthanasia**. **(A)** Cre = creatine/creatine phosphate, Lac = lactate, Myo = myoinositol, NAA = *N*-acetyl-aspartate, Gln = glutamine, Glu = glutamate, and Taur = taurine. **(B)** ADP = adenosine diphosphate, ATP = adenosine triphosphate, NAD = nicotinamide adenine dinucleotide, Ala = alanine, Asp = aspartate, Cho = choline, Gly = glysine, and GABA = gamma-amino butyric acid. PYR group differs from STD group at *p < 0.05 and **p < 0.01 (*t*-test).

The brains of APP/PS1 mice were snap frozen in liquid nitrogen at the end of the study and assessed with ^1^H-MRS. As summarized in Figure 5, levels of several energy metabolites, such as creatinine, glutamate, and lactate, were increased in the PYR group compared to STD group. Collectively, these findings support the idea that long-term dietary supplementation with pyruvate increases brain energy reserves.

## Discussion

The present study demonstrated that chronic dietary supplementation with pyruvate (PYR) in mice results in behavioral and metabolic changes in the brain. The pattern of behavioral outcomes was consistent between two independent experiments, one in 12-month-old wild-type mice and another in 6.5-month-old APP/PS1 mice. Long-term PYR supplementation (1) improved learning of the Morris swim task but did not significantly improve spatial memory as assessed by the search bias in the probe task, (2) enhanced preference to explore a novel odor, (3) increased rearing in a novel environment, and (4) robustly impaired performance in the passive avoidance task. The last effect could be observed in 5- to 6-month-old wild-type mice as well after chronic PYR supplementation but not after an acute post-training injection. A common denominator to all these effects is the enhanced exploratory activity, which is consistent with the observed increase in rearing in a new test cage in the PYR groups and increased running speed in a new environment in a set of controls studies. Finally, by using three different methods, we could detect increases in the brain energy metabolism after PYR supplementation confirming that even oral PYR administration can affect the brain energy status provided that the duration of the treatment is long enough.

### Improved Spatial Memory and Odor Recognition

The first behavioral finding, decreased escape latency in the Morris swim task by middle-aged wild-type mice on PYR supplementation, and a trend toward a better search bias in the probe task are in line with the recent findings in 3xTg mice showing improved spatial search bias by PYR treatment both at 6 and 12 months of age (Isopi et al., 2015). Unfortunately, Isopi et al. did not show the results of the acquisition phase in their report, making the direct comparison between the studies difficult. On the other hand, we did not find significant improvement in spatial search bias in 6.5-month-old APP/PS1 mice. Unfortunately, due to time and budgetary restrictions, we could not let our APP/PS1 mice age beyond 6.5 months of age when the spatial learning impairment is still modest. Thus, we cannot exclude the possibility that PYR supplementation would have been beneficial in 12-month-old APP/PS1 mice with more robust memory impairment. Why the effect was particularly pronounced during the acquisition phase and not during the probe test? Morris swim task is a very complex task and changes in many aspect of the behavior may lead to faster task acquisition. At least we could rule out the simple explanation that PYR worked against fatigue and slower swimming speed that would automatically lead to longer escape latencies. There was no difference in swimming speed among middle-aged wild-type mice overall, and importantly, not during the last trial of the day when the mice should have showed fatigue. It is worth noticing that we let the animals recover and warm up for 10 min between the trials to avoid hypothermia that easily develops in mice during swimming (Iivonen et al., 2003). Improved learning could also be seen in terms of decreased thigmotaxis, tendency for searching escape through the pool wall. This can be interpreted as improved cognitive flexibility (abandoning of an inefficient strategy) or decreased fear to move from the wall to the pool center. Together, these facts suggest that the treatment effects are more likely mediated by altered brain function rather than altered muscle function.

The odor recognition task has proven to be sensitive to age-dependent cognitive impairment in APP/PS1 mice (Koivisto et al., 2014). The task is based on the innate tendency of mice to explore a novel odor more than a familiar one. Not only should the mouse be familiar with its own odor but also to the presence of the wooden balls in its home cage during the familiarization phase. We cannot exclude the possibility that reduced exploration of a novel behaviorally meaningful odor stems from impaired olfaction, but in other tests, APP/PS1 mice have shown no impairment in habituation to the odor ball upon repeated exposures or learning to associate the odor of a natural extract to the presence of odorless food reinforcement. However, the test is not a pure recognition memory, either, since the outcome is also influenced by the interest to explore novelty vs. fear for an unknown smell. Interestingly, PYR supplementation led to significant improvement in the task in both middle-aged wild-type mice and adult APP/PS1 mice similar to our recent finding in middle-aged APP/PS1 mice after dietary supplementation with the omega-3 fatty acid DHA (Koivisto et al., 2014). However, the young adult APP/PS1 mice also showed a clear fear-related aversion toward the unknown conspecific on the first test day but no more on the second day. The most likely explanation for the pattern (which to a smaller extend could be seen in middle-aged wild-type mice as well) is that we see a combined effect of two factors: repetition-dependent fear for the unknown odors and PYR-induced increased tendency for exploration.

### Paradoxical Impairment in Passive Avoidance

The most consistent finding across age groups was the impairment in passive avoidance task. At first, it seems counterintuitive to see “memory improvement” in two behavioral tasks by PYR supplementation, but then a robust “memory impairment” in a third classic memory task. Further, an impaired memory consolidation would be unexpected, since both glucose (Gold, 2005) and lactate (Suzuki et al., 2011) given as post-training injections have been reported to enhance memory retention in this task. Consistent with this, we did not observe impairment after post-training injection of PYR at the dose shown effective in several previous studies (Lee et al., 2001; Suh et al., 2005; Isopi et al., 2015). However, passive avoidance is known to be a “quick and dirty” task with several behavioral confounds. We could rule out the possibility that PYR supplementation affects pain threshold. Decreased level of anxiety could easily mask the effect of fear conditioning and encourage the animal to enter the dark compartment. However, we did not find much evidence for PYR-induced anxiolysis. Although the marble burying task revealed a response that could be interpreted as anxiolytic, this was only seen in APP/PS1 mice and not in the middle-aged wild-type mice with PYR treatment. Further, the classic anxiety tests parameters, relative time spent on the open arms in the elevated plus maze or time in the center of the open field, were not influenced by PYR supplementation. The best explanation we can offer for the time being is that chronic PYR increased exploration of novelty, leading to the situation where the drive for exploration weighed more than the fear for the foot shock. In fact, increased exploration provides the best possible explanation to all present behavioral findings (less thigmotaxis and faster learning in the Morris swim task, increased exploration of a novel odor, increased rearing in a novel test cage, and increased running speed in the plus maze or open field during the first 2.5 min).

### Direct Pyruvate Effect or Secondary after Conversion to Lactate in the Liver?

The chow containing 6 g/kg pyruvate led to an average daily ingestion of 800 mg/kg/day after adjusting for food intake and spillage. Extrapolating to humans, these doses correspond to ~10 g daily pyruvate intake. A recent study showed that a single pyruvate dose of 0.1 g/kg resulted in a shift in fuel utilization toward accelerated carbohydrate oxidation lasting for several hours (Olek et al., 2015). In rodents, the neuroprotective effects of intraperitoneal pyruvate have been reported for doses ranging from 200 to 1000 mg/kg [for review, see Zilberter et al. (2015)]. The dose of pyruvate used in this study proved to be efficient; however, it is yet unclear what amount of pyruvate actually enters the brain at chronic oral administration. One can suggest that pyruvate may first be converted to lactate in the liver before it enters the brain. It has been shown, however, that glucose but not lactate is a preferred fuel for neurons *in vitro* (Patel et al., 2014), *ex vivo* (Ivanov et al., 2014), and *in vivo* in freely moving mice (Lundgaard et al., 2015). Meanwhile, the concentration of lactate in the extracellular cerebral fluid is close to that of glucose, and in the cortex is about 2–3 mM (Zilberter et al., 2010). Why then lactate is not utilized efficiently in spite of its relatively high level? We speculate that in the absence of extreme energy demands, the balance (presumably the redox state) is shifted toward glycolysis and subsequent release of excessive lactate from neurons (Dienel, 2012). In contrast, pyruvate is a “direct” energy substrate for mitochondria, while lactate needs to be converted first to pyruvate in the reaction dependent on the availability of cytoplasmic NAD^+^. In addition, besides being a mitochondrial fuel, pyruvate possesses a whole array of neuroprotective properties (Zilberter et al., 2015) lacking for lactate. A recent study on rats with Aβ microinfusion (i.c.v.) reported beneficial effects of pyruvate (500 mg/kg) after 10 daily intraperitoneal injections on spatial learning, LTP, ROS production, and neuronal survival. Corresponding effects were not obtained by systemic lactate injections, speaking for a direct pyruvate effect on neurons (Wang et al., 2015).

One may wonder why we did not find increased brain levels of pyruvate in the MRS assay. This is probably due to rapid pyruvate transformation to lactate. The kinetics of BBB transport of pyruvate is estimated to be 30- to 100-fold slower than the rate of pyruvate to lactate conversion. A recent MRI study employing hyperpolarized ^13^C-pyruvate could demonstrate the BBB penetration of a small bolus injection pyruvate and its conversion to lactate within 2 min in the brain (Hurd et al., 2010). A massive i.p. injection of 1000 mg/kg in an *in vivo* microdialysis study resulted in increased pyruvate levels in the plasma and interstitial fluid for 60 min, while the increased lactate levels were maintained for the entire 75-min follow-up time (Fukushima et al., 2009). Indeed, we could also detect increased levels of lactate in the brain, as well as those of glutamate and creatine/creatine phosphate. Considering that the mice were euthanized between 8:00 and 11:00 a.m. at least a couple of hours after significant ingestion of the chow during the dark period, it would have been impossible to even detect increased pyruvate levels in the circulation according to the microdialysis data. Thus, we must leave open the possibility that a fraction of pyruvate, when administered orally, may first be converted to lactate in the liver before it enters the brain.

In any event, we could confirm our previous finding that pyruvate as dietary supplement is able to increase brain glycogen levels (Zilberter et al., 2013). At this point, we cannot say whether the observed behavioral changes were directly linked to increased glycogen stores or whether increased brain glycogen only acts as a surrogate marker for improved brain energy status. Anyway, several neuronal processes have been shown to depend on astrocytic glycogen as their primary energy source. First, neurotransmitter glutamate is largely recycled, but ~15% is used for energy and needs to be replenished. This is mainly done by pyruvate carboxylase, which largely depends on glycogenolysis (Hertz et al., 2015). Second, the energy for Na^+^, K^+^-ATPase-mediated K^+^ uptake into astrocytes after prolonged neuronal activity derives from astrocytic glycogenolysis (Hertz et al., 2015). Third, inhibitor of glycogen phosphorylase inhibits memory consolidation in neonatal chicks (Gibbs et al., 2007) and rats (Newman et al., 2011; Suzuki et al., 2011).

### Indications for Pyruvate Supplementation

Besides emergency medicine, pyruvate has been extensively studied in sports medicine for its possible effects to improve muscle mass and endurance. Some studies have reported enhanced exercise endurance capacity by oral pyruvate combined with dihydroxyacetone (Stanko et al., 1990a,b) and enhanced fat loss when combined with low-energy diet (Stanko et al., 1992). However, in one study, the combination of pyruvate with creatine increased anaerobic performance while pyruvate supplementation alone was ineffective (Stone et al., 1999). Similarly, oral pyruvate alone for a week (Morrison et al., 2000) or in combination with creatine (Van Schuylenbergh et al., 2003) was ineffective to improve aerobic performance in cyclists, and 30-day puruvate supplementation failed to increase aerobic exercise performance in untrained subjects (Koh-Banerjee et al., 2005). Our findings in young adult mice are in line with these results, in that long-term pyruvate did not increase aerobic endurance or maximal anaerobic muscle force. At present, pyruvate supplements (creatine pyruvate) are actively marketed to body builders. The present study suggests a new indication for pyruvate by showing that dietary supplementation of pyruvate may offer a simple and effective way to correct for insufficient glucose supply and depleted glycogen stores in the aging brain. In addition to energy supplementation, pyruvate is a potent antioxidant, has anti-inflammatory, and anti-epileptic properties (Zilberter et al., 2015). It is well tolerated: up to millimolar plasma concentrations after i.v. infusion are reported not to have apparent adverse effects in humans (Dijkstra et al., 1984).

## Conclusion

Long-term dietary supplementation of pyruvate led to clear behavioral changes. Improved spatial learning, increased exploration of a novel odor, and a novel environment were seen in middle-aged wild-type mice as well as in adult APP/PS1 mice. The most consistent finding in all subgroups of mice was a shorter latency to enter the dark compartment in the passive avoidance task, which can be attributed to the increased drive for exploration. We found no evidence that pyruvate would act by changing muscle force or endurance. Increases in brain energy metabolites and glycogen stores after long-term pyruvate supplementation also speak for an effect on the brain metabolism. Dietary pyruvate supplementation may prove beneficial against aging-related cognitive impairment and inactivity.

## Author Contributions

HK: performed behavioral studies in middle-aged and APP/PS1 mice, analyzed the data, and collected all brain samples. HL: planned, performed, and supervised behavioral studies in young adult wild-type mice with chronic treatment, analyzed part of the data, and participated in writing the manuscript. MP and HH: performed behavioral studies in young adult wild-type mice with chronic treatment and analyzed the data. GB: set up PAS staining methods, performed histology on middle-aged mice, and analyzed the data. MS and HW: set up the analysis method for brain glycogen and ran the assay, analyzed the results, and participated in manuscript writing. MT and PS: ran all NMR spectroscopy assays, analyzed the results, and participated in writing the manuscript. YZ: planned the study with HT and major contribution in writing the manuscript. HT: PI of the whole study, supervision and coordination of all subprojects, and writing the manuscript.

## Conflict of Interest Statement

The authors declare that the research was conducted in the absence of any commercial or financial relationships that could be construed as a potential conflict of interest.
