# Corrigendum: Chronic Pyruvate Supplementation Increases Exploratory Activity and Brain Energy Reserves in Young and Middle-Aged Mice

**DOI:** 10.3389/fnagi.2017.00067

**Published:** 2017-03-20

**Authors:** Hennariikka Koivisto, Henri Leinonen, Mari Puurula, Hani S. Hafez, Glenda Alquicer Barrera, Malin H. Stridh, Helle S. Waagepetersen, Mika Tiainen, Pasi Soininen, Yuri Zilberter, Heikki Tanila

**Affiliations:** ^1^A. I. Virtanen Institute, University of Eastern FinlandKuopio, Finland; ^2^Biology Department, Faculty of Science, Suez UniversitySuez, Egypt; ^3^Institute of Physiology, Czech Academy of Sciences, v.v.i.Prague, Czechia; ^4^Faculty of Health and Medical Sciences, University of CopenhagenCopenhagen, Denmark; ^5^NMR Metabolomics Laboratory, School of Pharmacy, University of Eastern FinlandKuopio, Finland; ^6^Institute de Neurosciences des Systèmes, Aix Marseille Université, Institut National de la Santé et de la Recherche Médicale UMR_S 1106Marseille, France

**Keywords:** Alzheimer's disease, aging, memory, explorative activity, glycogen

In the Original Research article there was an error in the Section “Treatment” under the section “Methods” about the estimated daily intake of pyruvate:

“With the average food intake of 4 g this corresponds to 800 mg of pyruvate/day, which is at the upper range of effective pyruvate doses in earlier *in vivo* studies (Suh et al., 2005; Fukushima et al., 2009; Isopi et al., 2014).”

As correctly stated in the Abstract, the estimated dose was 800 mg of pyruvate/kg/day.

The corrected version of this section is shown below:

## Treatment

### Chronic pyruvate administration

The test group (PYR) received experimental chow supplemented with 0.6 % (w) of Na-pyruvate (Safe Diets, Augy, France). The control group (STD) received the same basic rodent chow (A04, Safe Diets). With the average food intake of 4 g this corresponds to 800 mg of pyruvate/kg/day, which is at the upper range of effective pyruvate doses in earlier *in vivo* studies (Suh et al., 2005; Fukushima et al., 2009; Isopi et al., 2014). *Acute pyruvate administration*. The mice received Na-pyruvate (Sigma, St. Louis, MO, USA) 500 mg/kg i.p. or the same molar concentration of NaCl (260 mg/kg i.p.). This single dose affords neuroprotection against cortical concussion injury and increases brain glucose and pyruvate levels as measured by *in vivo* microdialysis (Fukushima et al., 2009). All cage labels about the treatment groups were coded so that the researchers running behavioral tests or assays on post-mortem samples were blinded as to the treatment.

### Conflict of interest statement

The authors declare that the research was conducted in the absence of any commercial or financial relationships that could be construed as a potential conflict of interest.

